# Rapid Uptake of the Subcutaneous Injectable in Burkina Faso: Evidence From PMA2020 Cross-Sectional Surveys

**DOI:** 10.9745/GHSP-D-17-00260

**Published:** 2018-03-21

**Authors:** Georges Guiella, Shani Turke, Hamadou Coulibaly, Scott Radloff, Yoonjoung Choi

**Affiliations:** a Institut Supérieur des Sciences de la Population (ISSP), Université Ouaga 1 Pr Joseph Ki-Zerbo, Ouagadougou, Burkina Faso.; b Bill & Melinda Gates Institute for Population and Reproductive Health, Department of Population, Family, and Reproductive Health, Johns Hopkins Bloomberg School of Public Health, Baltimore, MD, USA.

## Abstract

The subcutaneous (SC) injectable, widely known by its commercial name Sayana Press, has potential to improve access to contraceptive methods. In Burkina Faso, SC-injectables were first piloted in select regions in 2014 and introduced nationally in 2016. PMA2020 is the first national survey to track programmatic progress of SC-injectable introduction at both population and health facility levels in the country across 2 rounds of data collection: March–May 2016 and November 2016–January 2017. Over this 6-month period, SC-injectable availability at public service delivery points increased from 50% to 85%, largely driven by increases in availability among the non-pilot regions. In terms of use, while the modern contraceptive prevalence rate among all women remained constant at about 23%, SC-injectable prevalence nearly doubled from 1.1% to 2.0%, making up approximately 9% of all modern method users in Burkina Faso by late 2016, though the difference was not statistically significant. Increases were comparable between pilot and non-pilot regions. While the difference was not statistically significant, more rural women were using the method compared with their urban counterparts in the pilot regions, an interesting finding considering the opposite pattern is generally true for contraceptive prevalence nationally. In summary, following national scale-up, data show substantially improved availability of SC-injectables at service delivery points and potential for changes in the method mix in Burkina Faso. In order to further improve contraceptive access and choice, scale-up of community-based distribution of SC-injectables should be considered, especially among rural populations with higher unmet need for family planning.

## Abstract (precis)

Availability and use of the subcutaneous injectable increased rapidly during national scale-up in 2016. Substantial increases were found in rural areas, where unmet need for family planning is higher. Since the method is amenable to community-based distribution, a new pilot is testing provision by community health workers to further improve access.

## INTRODUCTION

The subcutaneous (SC) injectable, often referred to by its commercial name Sayana Press, offers a new delivery design for injectable contraceptives that uses a simplified autodisable injection system (Uniject) to administer a lower dose of depot medroxyprogesterone acetate (DMPA). The simplified delivery mode allows providers at lower levels of the health system, such as community health workers (CHWs), and potentially users themselves to administer the method, with the ultimate goal of increasing women's access to a wider range of contraceptive methods.^1^^–^^4^ Studies conducted in Senegal and Uganda have demonstrated the method's acceptability and have identified approaches for its safe and effective introduction.^1^^–^^3^ Since then, SC-injectables have been approved for use in a number of countries in Africa and Asia. A recent price reduction making the unit cost comparable to that of the intramuscular (IM) injectable will certainly also help improve affordability and access.^5^

As part of its larger strategy under the Ouagadougou Partnership and its Family Planning 2020 (FP2020) commitments to increase women's access to modern contraceptive methods, the government of Burkina Faso issued regulatory approval for SC-injectables in 2013. In 2014, the Direction de la Santé de la Famille (DSF), a division of the Burkinabé Ministry of Health, piloted SC-injectable introduction in 4 of 13 regions, focusing on regions with higher rates of unmet need for family planning^6^: Boucle du Mouhoun, Centre, Centre-Ouest, and Hauts-Bassins. The pilot program introduced SC-injectables in communities as well as facilities, intending to address both demand generation at the population level and supply factors at the facility level. From the outset, it was designed to demonstrate approaches to be readily scaled up at the national level.^7^ Evaluation of program implementation confirmed the importance of key components that were initially designed in the pilot program, including supply chain management and training of health workers for better counseling, and the evaluation recommended no major changes in the approach for scale-up.

National scale-up beyond the 4 pilot regions occurred in 2016, again focusing on both demand generation and service provision. All components demonstrated during the pilot program were replicated in the remaining 9 regions. The DSF implemented introduction activities with technical and financial support from PATH, the United Nations Population Fund (UNFPA), and the Deutsche Gesellschaft für Internationale Zusammenarbeit (GiZ) among various other national and international NGOs. The pilot program is described in further detail below.

Burkina Faso piloted introduction of the subcutaneous injectable in 2014 in 4 of 13 regions, and scaled up nationally in 2016 to the other 9 regions.

No nationally representative studies have assessed availability and uptake of SC-injectables since their introduction in Burkina Faso. This study aims to assess such progress, using 2 rounds of data from Performance Monitoring and Accountability 2020 (PMA2020), a nationally representative survey platform for rapid performance monitoring. Specific objectives of this article are to explore trends in service and commodity availability for SC-injectables at facilities, as well as SC-injectable use at the population level, and assess differential uptake among women by background characteristics. The study provides for the first time national-level evidence on programmatic progress and population-level use of SC-injectables.

## SC-INJECTABLES PILOT PROGRAM

**FIGURE 1.**
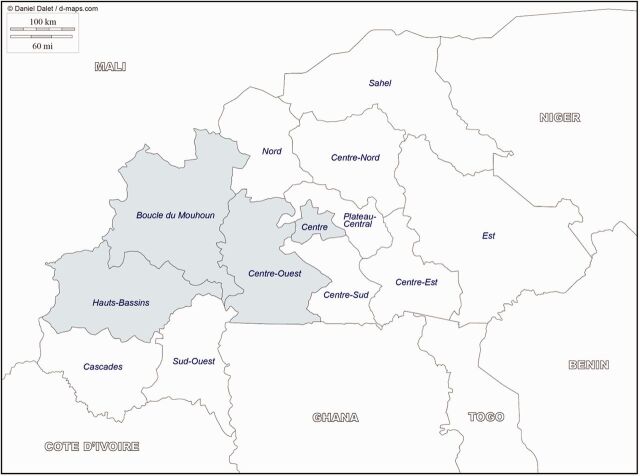
Four Regions for the Pilot Program Introducing Subcutaneous Injectables

The pilot program introduced SC-injectables in 4 selected contiguous regions of Burkina Faso with relatively high unmet need for family planning: Boucle du Mouhoun, Center, Center-Ouest, and Hauts-Bassins, as shown in Figure 1.^7^ Launched in July 2014, the program aimed to improve the range of contraceptive methods available to women in order to accelerate increases in modern method use. By the end of 2015, the pilot aimed to train 700 providers on SC-injectable administration, ensure availability of the method in all public primary and district-level health facilities in these regions, strengthen communication campaigns and social marketing of the method, and augment the total number of additional modern method users. The program employed 2 coordinated strategies: (1) service provision improvement at facilities, and (2) demand generation for the method among the target population.

Through provider training and direct method provision, SC-injectables were introduced in all public health facilities in the 4 regions, consisting of 23 district health centers and 684 primary health facilities, or Centres de Santé et de Promotion Sociale (CSPS). In addition, through a public-private partnership with Marie Stopes International (MSI) and the Burkina Faso Family Welfare Association (ABBF), the method was offered at 8 additional private, nonprofit facilities managed by the organizations as well as their 7 mobile teams. The mobile teams were designed especially to reach women in geographically inaccessible and remote areas. Of the 250,000 doses of SC-injectables made available by the donor consortium in early 2014, 72,500 doses were sent to these service delivery points (SDPs) as initial stocks for immediate service provision, once providers were trained.

A cascading approach was used to train providers at multiple levels. The program trained higher-level providers (i.e., doctors, specialized nurses/midwives, and other health professionals) to administer the method first. They, in turn, trained nurses at CSPS and at clinics managed by NGOs. No other lower-level health workers such as CHWs were authorized or trained to provide the method because the policy environment did not allow community-based distribution of injectable contraceptives. However, an alternative strategy was used to improve access to the method at the community level: family planning services, including SC-injectables, were integrated with vaccination outreach campaigns conducted by the Expanded Programme on Immunization (EPI) in villages. A total of 1,908 providers were trained, including 527 itinerant EPI health workers to ensure the integration of SC-injectables with immunization outreach.

CHWs were not authorized or trained to provide the subcutaneous injectable because the policy environment did not allow community-based distribution of injectables.

In addition, demand for the method was generated using mass media campaigns and interpersonal and community-based communication activities. Mass media campaigns consisted of production and broadcasting of TV and radio messages among the most popular media channels in rural areas. The campaigns also produced print materials, including posters, brochures, and leaflets, which were distributed to the general population at facilities. Second, a more targeted information, education, and communications (IEC) approach was carried out. For example, *Boîtes à Images* (“Image Boxes,” or a small collection of boxed flash cards with simple images depicting the SC-injectable and how it is administered) to promote SC-injectables were developed and used at health facilities across all levels, from CSPS up to Regional Health Directorates and managing authorities. A promotional film on SC-injectables was also produced and distributed to Regional Directorates of the Ministry of Health, NGO partners, and some youth associations, in order to help promote the new product. Community-based communication activities for demand generation included an innovative strategy called “Family Planning Djandjoba” or *laafi rââga* (health promotion market day in Mooré, the main local language in Burkina Faso), which mobilized women for a popular dance in the villages. During the event, family planning messages were disseminated, and women were given the opportunity to adopt family planning methods discreetly. These strategies may have generated greater interest in SC-injectable use among rural women.

Importantly, IEC facilitators, CHWs, and other community leaders were trained to lead demand generation outreach sessions at the community level in rural areas. While CHWs were not authorized to administer the method as previously mentioned, CHWs were mobilized to generate demand for the method.

## METHODS

### Data

PMA2020 is a survey platform relying on resident enumerators to collect data on smartphones in order to track progress toward FP2020 goals, which were pledged by countries following the 2012 London Summit on Family Planning. Data are collected every 6 months during the first 2 years in a country, and annually thereafter. For each round, surveys are conducted among populations and at SDPs. The population-based survey collects data from a nationally representative sample of households, selected using a 2-stage cluster sampling design. The survey sample size is determined to estimate the modern contraceptive prevalence rate with a 3% margin of error at the national level. Household interviews are conducted with a competent household member, often the household head, and information is collected on demographic characteristics of all household members as well as household characteristics. Interviews are also conducted with all women 15–49 years of age in sampled households to collect additional information on background characteristics, fertility preferences, demand for family planning, and contraceptive use.

The SDP survey is conducted among both public and private SDPs that are geographically and administratively accessible to the sampled population. Facility data are collected to measure availability of contraceptive methods among the primary, secondary, and tertiary public facilities designated to serve each sampled enumeration area (EA), and up to 3 private facilities located within the EA. Further information on survey design and implementation is available elsewhere.^8^^,^^9^

Burkina Faso has conducted 4 rounds of PMA2020 surveys since 2014. To monitor the impact of SC-injectable introduction, the third and fourth rounds (conducted in March–May 2016 and November 2016–January 2017, respectively) included questions on SC-injectable use in the female questionnaire. Among women who reported current injectable use, a follow-up question was asked: “Was the injection administered via syringe or small needle?” Enumerators probed the respondent using images of the syringe and Uniject delivery modes on their smartphone screen. A total of 3,261 and 3,195 women were interviewed in Rounds 3 and 4, respectively.

In the SDP questionnaire, SC-injectables were included in the list of contraceptive methods to measure service availability (i.e., if a specific method was provided at facilities), availability of the method on the day of survey (verified by observation), and 3-month history of stock-outs. The SDP sample included 132 and 131 facilities in Rounds 3 and 4, respectively. Public facilities made up over 80% of the SDP sample, reflecting the extent and geographic distribution of private facility coverage in the country.

### Measurement

A woman was categorized as an SC-injectable user if she reported using injectables *and* a small needle was used to administer the method. To assess any differential uptake among women, background characteristics were assessed including current union status (i.e., married or cohabiting vs. not in union), region (4 initial pilot regions vs. 9 non-pilot regions), residential area (urban vs. rural), and household wealth tertile, based on a wealth index.^10^ Finally, using the SDP surveys, binary variables were constructed to measure whether facilities provided SC-injectables and whether the method was currently available at the facility.

### Analysis

Analyses were conducted using data from both the female and SDP surveys in Rounds 3 and 4. Rounds 1 and 2 did not include SC-injectable questions and were thus not included in this analysis. To measure trends of availability of SC-injectables at SDPs, we estimated the percentage of facilities providing the method and percentage of facilities providing the method *and* having it in stock on the day of survey, by survey round. In order to compare it against availability of IM-injectables, corresponding estimates were calculated.

To assess trends in SC-injectable use, we calculated the percentage of women using any method; any modern method (i.e., male or female sterilization, the intrauterine device [IUD], implants, SC- or IM-injectables, pills, male or female condoms, Lactational Amenorrhea Method, emergency contraception, diaphragm, spermicide, or the Standard Days Method); and SC-injectables in each round. Additionally, we compared distributions of methods among modern method users across the 2 survey rounds. The percentage of women using SC-injectables was assessed further by background characteristics, to explore any differential uptake. All estimates based on female interview data were adjusted for sample design, and 95% confidence intervals (CIs) were calculated. We used STATA 14.2 for all analyses.

## RESULTS

Only 50% of public facilities offered SC-injectables in March–May 2016, compared with 98% for IM-injectables (Table 1). By late 2016, however, SC-injectables were offered in 85% of public facilities. The increase over 6 months came largely from the 9 regions where the method was introduced in 2016, with an increase from 20% to 81%, compared with 84% to 91% in the initial pilot regions. By late 2016, method availability was high among public facilities offering SC-injectables: 98% in the 4 pilot regions and 80% in the 9 non-pilot regions. The changes among all facilities mirror those among public facilities, as public facilities consist of nearly 90% of all facilities in the country.

**TABLE 1.** Percentage of Facilities Offering Intramuscular and Subcutaneous Injectables and With the Commodity in Stock Among Pilot and Non-Pilot Regions, PMA2020 Survey Rounds 3 and 4

	Round 3			Round 4		
	March–May 2016			November 2016–January 2017		
	All Regions	In the 4 Pilot Regions	In the 9 Non-Pilot Regions	All Regions	In the 4 Pilot Regions	In the 9 Non-Pilot Regions
**All facilities**						
Number of facilities	132	62	70	131	61	70
Offer IM-injectables, %	84.1	83.9	84.3	91.6	90.2	92.9
Offer IM-injectables *and* the method is in stock, %	83.3	83.9	82.9	90.8	88.5	92.9
Offer SC-injectables, %	42.4	69.4	18.6	76.3	78.7	74.3
Offer SC-injectables *and* the method is in stock, %	39.4	64.5	17.1	67.9	77.0	60.0
**Public facilities**						
Number of facilities	110	51	59	116	53	63
Offer IM-injectables, %	98.2	100.0	96.6	100.0	100.0	100.0
Offer IM-injectables *and* the method is in stock, %	97.3	100.0	94.9	100.0	100.0	100.0
Offer SC-injectables, %	50.0	84.3	20.3	85.3	90.6	81.0
Offer SC-injectables *and* the method is in stock, %	46.4	78.4	18.6	75.9	88.7	65.1

Abbreviations: IM, intramuscular; PMA2020, Performance Monitoring and Accountability 2020; SC, subcutaneous.

Availability of the subcutaneous injectable increased in public facilities, from 50% in early 2016 to 85% in late 2016.

**FIGURE 2.**
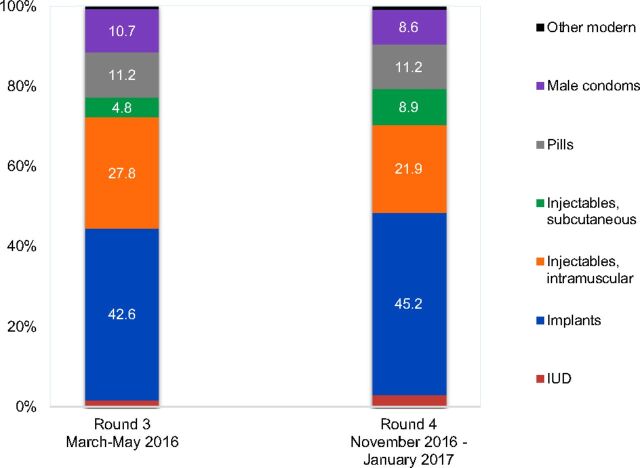
Percentage Distribution of Contraceptive Methods Among All Modern Method Users, PMA2020 Survey Round 3 and Round 4 Abbreviations: IUD, intrauterine device; PMA2020, Performance Monitoring and Accountability 2020.

The level of modern contraceptive use remained comparable over 6 months, at about 23% among all women (Table 2). The percentage of all women using SC-injectables, however, increased from 1.1% (95% CI: 0.7, 1.7) to 2.0% (95% CI: 1.3, 3.0). Although not statistically significant, data also showed an increased share of SC-injectables among women using modern methods, from 4.8% in March–May 2016 (95% CI: 3.5, 6.4) to nearly 9% (95% CI: 6.9, 11.5) by late 2016 (Figure 2). Among unmarried sexually active women who use modern methods, the relative contribution of SC-injectables remained low.

**TABLE 2.** Modern Contraceptive Use Among All Women, Women in Union, and Unmarried Sexually Active Women, PMA2020 Survey Rounds 3 and 4

Percentage Using	Round 3			Round 4		
	March–May 2016			November 2016–January 2017		
	All Women (N=3261)	Women in Union (n=2239)	Unmarried Sexually Active Women (n=218)	All Women (N=3196)	Women in Union (n=2220)	Unmarried Sexually Active Women (n=226)
Any method	22.8	25.5	43.5	22.9	25.5	41.6
Any modern method	21.5	24.2	41.2	22.0	24.5	38.8
IUD	0.4	0.5	0.0	0.7	0.9	0.5
Implants	9.2	10.9	12.8	9.9	11.8	7.1
IM-injectables	6.0	7.5	5.9	4.8	5.7	8.1
SC-injectables	1.1	1.3	0.3	2.0	2.5	1.0
Pills	2.4	2.8	4.7	2.5	2.8	5.0
Male condoms	2.3	1.0	17.1	1.9	0.8	16.4
Other modern method	0.2	0.2	0.3	0.2	0.1	0.6

Abbreviations: IM, intramuscular; IUD, intrauterine device; PMA2020, Performance Monitoring and Accountability 2020; SC, subcutaneous.

The percentage of all women using subcutaneous injectables increased from 1.1% to 2.0% over a 6-month period in 2016.

Table 3 shows the increased use of SC-injectables across various background characteristics. Use increased in both the initial pilot regions, from 1.7% (95% CI: 1.1, 2.5) to 2.6% (95% CI: 1.5, 4.6), and in the non-pilot regions, from 0.7% (95% CI: 0.3, 1.9) to 1.5% (95% CI: 0.8, 2.8), although use was still higher in pilot regions. Although not statistically significant, SC-injectable use in late 2016 was slightly higher among rural women compared with their urban counterparts, 2.1% (95% CI: 1.2, 3.5) vs. 1.7% (95% CI: 1.0, 2.8), respectively; an occurrence further pronounced among rural women in pilot regions, where SC-injectable use increased from 1.8% (95% CI: 1.0, 3.4) to 3.2% (95% CI: 1.3, 7.2) (Table 4). There was no clear pattern of SC-injectable use by wealth among women in the pilot regions. However, among women in non-pilot regions, a clearer wealth gradient was reported, with 4.0% (95% CI: 1.9, 8.1) of the wealthiest women using SC-injectables, compared with 0.7% (95% CI: 0.2, 2.3) among the poorest.

**TABLE 3.** Percentage of All Women Reporting Use of Subcutaneous Injectables by Background Characteristics, PMA2020 Survey Rounds 3 and 4

	Round 3			Round 4		
	March–May 2016			November 2016–January 2017		
	n	%	95% CI	n	%	95% CI
**Total**	3268	1.1	(0.67, 1.72)	3213	2.0	(1.28, 2.99)
**Region**						
4 pilot regions	1281	1.7	(1.09, 2.54)	1389	2.6	(1.45, 4.61)
9 non-pilot regions	1987	0.7	(0.25, 1.91)	1824	1.5	(0.78, 2.77)
**Residential area**						
Urban	783	1.1	(0.58, 2.06)	759	1.7	(1.00, 2.81)
Rural	2484	1.1	(0.58, 1.94)	2454	2.1	(1.21, 3.45)
**Household wealth index**						
Highest tertile	1098	1.4	(0.80, 2.29)	1058	2.7	(1.69, 4.41)
Middle tertile	996	0.7	(0.28, 1.56)	1044	2.0	(0.99, 4.07)
Lowest tertile	1174	1.2	(0.55, 2.42)	1111	1.2	(0.54, 2.49)

Abbreviations: CI, confidence interval; PMA2020, Performance Monitoring and Accountability.

**TABLE 4.** Percentage of All Women Reporting Use of the Subcutaneous Injectable by Background Characteristics, PMA2020 Survey Rounds 3 and 4

	Round 3			Round 4		
	March–May 2016			November 2016–January 2017		
	n	%	95% CI	n	%	95% CI
**4 PILOT REGIONS**						
**Residential area**						
Urban	565	1.5	(0.76, 2.77)	557	1.7	(0.90, 3.20)
Rural	716	1.8	(0.98, 3.41)	832	3.2	(1.32, 7.24)
**Household wealth index**						
Highest tertile	655	1.5	(0.83, 2.83)	688	2.1	(1.24, 3.49)
Middle tertile	358	1.0	(0.31, 3.40)	383	3.7	(1.29, 10.06)
Lowest tertile	268	2.8	(1.13, 6.85)	318	2.4	(0.93, 6.18)
**9 NON-PILOT REGIONS**						
**Residential area**						
Urban	218	0.2	(0.02, 1.52)	203	1.6	(0.60, 4.29)
Rural	1769	0.8	(0.26, 2.18)	1622	1.5	(0.70, 3.00)
**Household wealth index**						
Highest tertile	443	1.1	(0.35, 3.36)	370	4.0	(1.91, 8.07)
Middle tertile	638	0.5	(0.11, 1.75)	661	1.1	(0.47, 2.36)
Lowest tertile	906	0.7	(0.20, 2.20)	793	0.7	(0.18, 2.34)

Abbreviations: CI, confidence interval; PMA2020, Performance Monitoring and Accountability 2020.

## DISCUSSION

SC-injectables were first introduced in Burkina Faso in 4 pilot regions in 2014, with both demand generation and supply of the methods at facilities being addressed. The introduction program was designed ready for scale-up, and pilot evaluation recommended replication without major modifications. Thus, the same introduction program was scaled up nationally in 2016. As a result, SC-injectable availability increased substantially over a 6-month period during 2016. Although availability remained higher in pilot regions, by the end of 2016 SC-injectables were provided in 85% of public facilities accessible to the national population.

Results indicate SC-injectable use has likely increased over the same period, although the difference was not statistically significant at the 95% confidence level. The percentage of women using SC-injectables almost doubled, from 1.1% to 2.0% of all women, accounting for 9% of the modern method mix. Similar to the facility data, SC-injectable use remained higher in pilot regions, but the rate of increase was similar between pilot and non-pilot areas. While the magnitude of these changes did not reach statistical significance—largely since sample sizes for the survey were determined to monitor the overall modern contraceptive prevalence rate—the direction of changes in use is likely valid, as it is consistent and corroborated by implementation strategies and availability data from SDPs in our study.

Although not statistically significant, SC-injectable use was higher in rural than urban areas, which is interesting considering overall contraceptive prevalence is higher in urban areas in the country.^11^^,^^12^ This may have been a result of SC-injectable demand generation strategies that relied on CHWs, targeting rural rather than urban zones. Some health districts mobilized CHWs to promote SC-injectables, although they were not authorized to administer the method. For example, in Reo and Orodara districts, located in the pilot regions, CHWs could offer SC-injectables for purchase so that the method could be administered during outreach events or at a woman's visit to a facility.

Given the cross-sectional design of PMA2020 surveys, we are unable to determine if SC-injectable users switched from other contraceptive methods or were entirely new contraceptive users. Considering comparable contraceptive prevalence rates coupled with changes in method mix between the 2 rounds, we speculate that SC-injectables were likely adopted by women already using a method, probably the IM-injectable. This would make sense given that the initial SC-injectable introduction occurred only among facilities already authorized to provide IM-injectables, without fully capitalizing on the elements of the SC-injectable that distinguish it from the IM-injectable.

Recently, community-level distribution of SC-injectables has begun in 2 pilot districts: Dandé in Hauts-Bassins and Tougan in the Boucle du Mouhoun. Based on a developed task-shifting strategy, 128 CHWs already working at CSPS in the 2 districts were trained between October 2016 and March 2017 to provide oral contraceptive pills and the SC-injectable. Service provision by CHWs started in May 2017, after a follow-up training and supervision at the end of April 2017. This expanded service delivery strategy and the recent price reduction is expected to further improve access to SC-injectables and, ultimately, choice and overall use of contraceptive methods among women in rural areas who have higher unmet need.^11^^,^^12^

Community-level provision of the subcutaneous injectable by CHWs has recently begun in 2 pilot districts in Burkina Faso.

It is also notable that SC-injectable use remained minimal among unmarried sexually active women, despite IM-injectable use being common among this population. This may imply that SC-injectable introduction did not address barriers specific to unmarried adolescents such as provider bias^13^ and/or that the introduction efforts explicitly focused on women in union.

### Limitations

PMA2020's sample design does not allow detection of statistical significance for method-specific contraceptive prevalence rates nationally. We acknowledge large sampling error as the surveys are not designed to measure changes in method-specific use with statistical significance, but the direction of the trend is further supported by SDP-level data and corroborating programmatic efforts in the country. Finally, awareness of SC-injectables or switching of contraceptive methods was not assessed in either round. Considering SC-injectable demand generation campaigns in the country, inclusion of awareness questions should be considered in future survey rounds. Regarding method switching, PMA2020 is currently testing and considering adding questions to measure method switching in future surveys. Such information will provide important programmatic insight, especially in populations where new methods are introduced and promoted.

## CONCLUSION

In conclusion, in Burkina Faso, SC-injectables were introduced in pilot regions in 2014, and the program was scaled up nationally in 2016. We report rapid increases in SC-injectable availability and use over 6 months in 2016 using nationally representative survey data. Community-level distribution by CHWs currently being piloted may further improve access to the method in rural settings. Routine and rapid monitoring surveys, such as PMA2020, can continue to provide critical data for tracking nationally scaled programmatic progress at both the population level and among health facilities.

## Notes

**Peer Reviewed**

**First Published Online:** January 25, 2018

**Funding:** PMA2020 is implemented by the Bill & Melinda Gates Institute for Population and Reproductive Health at the Johns Hopkins Bloomberg School of Public Health, with generous support by the Bill & Melinda Gates Foundation (grant #OPP1079004).
